# Supplementary figures and images for: Seizure epicenter depth and translaminar field potential synchrony underlie complex variations in tissue oxygenation during ictal initiation
Source: Neuroimage. 2018 May 1;171:165–75. doi: 10.1016/j.neuroimage.2017.12.088 (PMC5883323; doi:10.1016/j.neuroimage.2017.12.088)

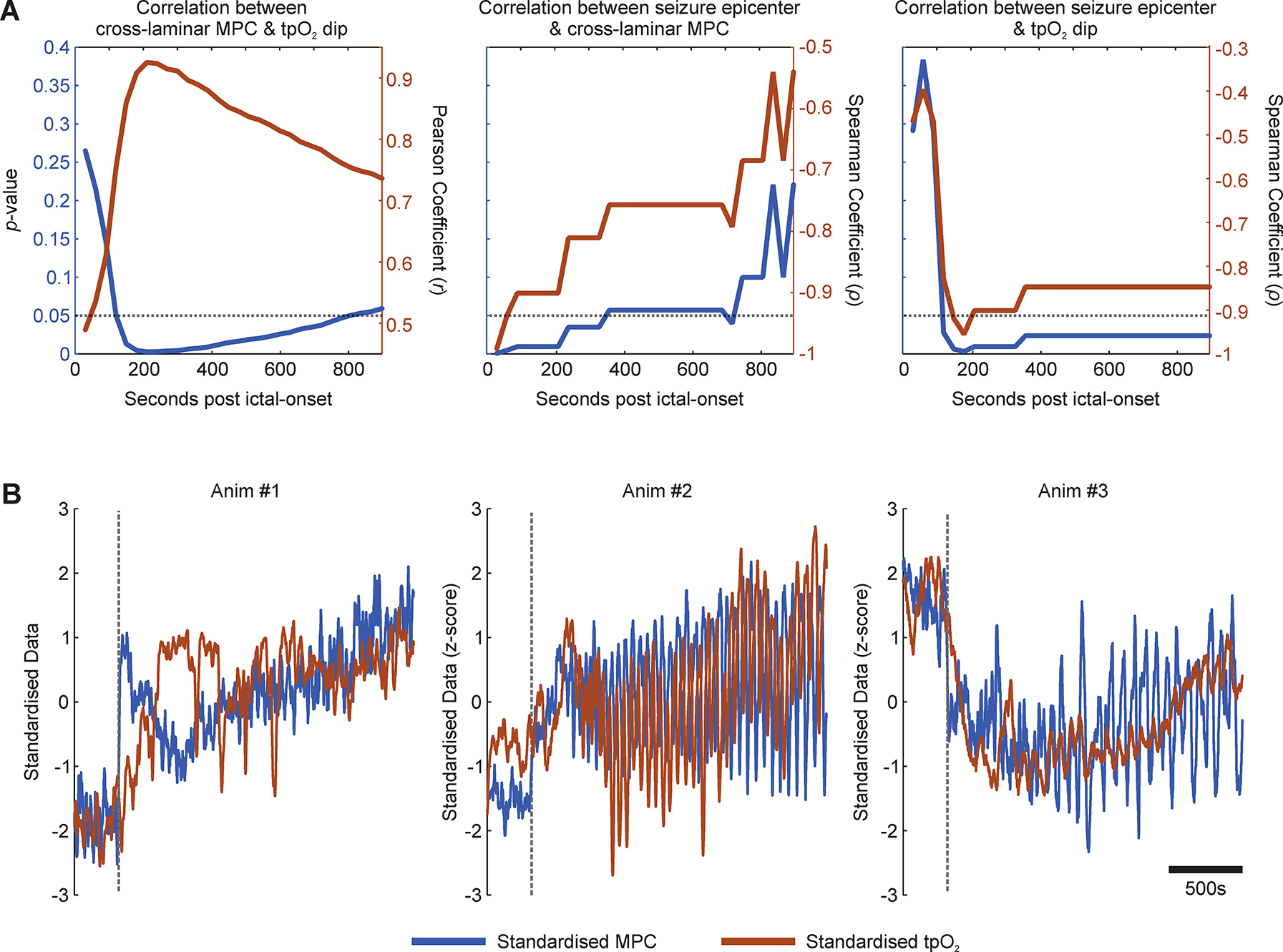

Supplement: Supplementary material [file figs1.jpg]
